# Validation of the Seattle Angina Questionnaire in patients with myocardial infarction

**DOI:** 10.1097/MD.0000000000047546

**Published:** 2026-01-30

**Authors:** Ali Albarrati, Taghreed Alotaibi, Rakan Nazer

**Affiliations:** a Rehabilitation Sciences Department, College of Applied Medical Sciences, King Saud University, Riyadh, Saudi Arabia; b Prince Sultan Cardiac Center, Prince Sultan Military Medical City, Riyadh, Saudi Arabia; c Cardiac Sciences Department, College of Medicine, King Saud University, Riyadh, Saudi Arabia.

**Keywords:** MI, quality of life, reliability, SAQ-7, validity

## Abstract

The short version of the Seattle Angina Questionnaire-7 (SAQ-7) is widely used in patients with coronary artery disease, but its use in patients post myocardial infarction (MI) is limited. This study aimed to validate the SAQ-7 in patients post-MI. A longitudinal cohort study was conducted with 258 stable participants with MI recruited from different outpatient cardiology clinics. Participants with cardiologist-confirmed MI, stable ≥4 weeks were included in the study, while participants with ejection fraction <40%, neurological or musculoskeletal disorders were excluded. Participants completed the SAQ-7, European quality of life-five dimensions-five levels (EQ-5D-5L), and Short Form 12 (SF-12) at baseline. Questionnaires were self-administered, and assistance was given when required. A subset of 117 participants returned 3 to 10 days later for a follow up to assess test-retest reliability. Participants completed the SAQ-7 alongside with a global rating of change scale (GRC). Cronbach alpha and intraclass correlation coefficient (ICC_2,1_) were used to examine internal consistency and test-retest reliability, respectively. Construct validity was determined via correlations with EQ-5D-5L and SF-12 scores. The SAQ-7 demonstrated excellent internal consistency (Cronbach alpha = 0.97) and test-retest reliability (ICC_2,1_ = 0.94; 95% CI: 0.92–0.96, *P* <.001). The SAQ-7’s measurement error was 1.76, with a minimum detectable change at 95% confidence of 4.87 points. The Bland and Altman analysis showed a mean difference of 0.75 with narrow limits of agreement. The SAQ-7 correlated strongly with EQ-5D-5L (r = -0.86, *P* <.001) and moderately with the SF-12 Physical (*R* = 0.74, *P* <.001) and Mental (*R* = 0.57, *P* <.001) components scores. No floor or ceiling effects were observed. The SAQ-7 demonstrated excellent psychometric properties in post-MI, supporting its use for health related quality of life assessment. However, these findings should be interpreted in the context of the study population and design.

## 1. Introduction

Myocardial infarction (MI) is a major medical condition linked to decreased physical activity, reduced social participation, and an increased risk of depression.^[1,2]^ A key consequences of MI is decline in health related quality of life (HRQOL).^[3]^ The influence of MI on an individual’s HRQOL is a critical aspect that needs to be considered during evaluation and medical interventions.^[4]^ HRQOL is a multifaceted concept that includes patient’s symptoms, functional capacity and psychological health, and it serves as a crucial determinant in guiding therapeutic decision making.^[4–7]^

Assessment of HRQOL in MI can be performed using either generic or disease-specific patient-reported outcome measures (PROMs). Generic instruments such as the EQ-5D-5L and SF-12 are widely applied in post-MI populations, and provide a comprehensive view of overall health, and enable comparison across different clinical conditions.^[8–11]^ While these tools capture overall health and the total burden of MI, they may lack the sensitivity to detect specific angina-related issues or changes in symptoms.^[12]^

The SAQ is a disease-specific HRQOL questionnaire designed for patients with coronary artery disease (CAD).^[13]^ The original SAQ was developed in 1994 and consists of a 19-item to assess the health outcomes in patients with CAD across 5 domains: physical limitation, angina stability, angina frequency, disease perception, and treatment satisfaction.^[13]^ It has demonstrated validity, reliability, and sensitivity in patients with CAD.^[13,14]^ Although the original SAQ has been widely used in clinical trials and registries, its use in routine clinical practice has been limited due to its length. To address this, the SAQ-7 was developed and validated against the original version, and shown to preserve the full instrument’s psychometric and prognostic properties.^[15]^ The SAQ-7 is a validated scale, and sensitive to clinical changes in patients with CAD.^[16–19]^ Although the SAQ-7 is more widely used in clinical studies in patients with chronic CAD, its application in patients post-MI is still limited. Therefore, this study aimed to test the measurement properties of the SAQ-7 in patients post-MI.

## 2. Materials and methods

### 2.1. Study design and setting

The study was a cohort longitudinal study involving participants with MI from multi cardiac center in Saudi Arabia.

### 2.2. Sample size

In accordance with consensus-based standards for selecting health measurement instruments (COSMIN), the sample size was recommended to be a minimum of 100 participants, which is considered an adequate sample size for testing consistency, test-retest reliability, and construct validity.^[20]^ This study received ethical approval from the Institutional Review Board (IRB Number: E-22-129E) of King Saud University, Riyadh, Saudi Arabia.

### 2.3. Participant

Adult participants with cardiologist-confirmed MI, and were stable for at least 4 weeks post-MI were included in this study. Participants with ejection fraction below 40%, had neurological disorders, or severe musculoskeletal diseases that directly impact HRQOL were excluded. Questionnaires were self-administered in cardiac outpatient settings, and assistance were given when required. Participants reporting significant health changes between the visits were excluded from reliability analysis.

### 2.4. Procedures

#### 2.4.1. *Testing the psychometric properties of the SAQ*

Eligible participants provided informed consent at their first visit, completed demographic information, and filled out the SAQ-7, EQ-5D-5L, and SF-12 questionnaires. A follow-up visit was scheduled 3 to 10 days later to assess test-retest reliability of the SAQ-7.^[21]^ Before completing the SAQ-7 during the follow-up, participants were asked to report any changes in health status since the initial visit by completing the GRC questionnaire.

### 2.5. *Outcome measures*

#### 2.5.1. The Seattle Angina Questionnaire-7

The SAQ-7 is a validated tool that assesses 3 dimensions of HRQOL in patients with CAD: physical limitation, angina frequency, and quality of life. For each domain, scores ranged from 0 to 100. Furthermore, an overall summary SAQ-7 score was calculated by averaging the 3 domain scores, with higher scores indicating less frequent angina, better function, and better quality of life.^[16]^ The SAQ-7 Summary score, SAQ-7 Physical Limitation score, and SAQ-7 Quality of Life score was classified into ranges of 0 to 24 (very poor to poor health status), 25 to 49 (poor to fair), 50 to 74 (fair to good), and 75 to 100 (good-to-excellent health status). The SAQ-7 Angina Frequency scores were classified according to the severity of angina: a score 0–30 indicates daily angina, a score = 31 to 60 indicates weekly angina, monthly angina is scored between 61 to 99, and a score of 100 represents no angina.^[16]^

#### 2.5.2. European quality of life-five dimensions-five levels

The EQ-5D-5L is a generic measure of HRQOL, which has been developed by Europe QOL group. The EQ-5D-5L consists of a descriptive system. The descriptive system evaluates 5 dimensions of health, including mobility, self-care, usual activities, pain/discomfort, and anxiety/depression. Each of the 5 items in the EQ-5D-5L is rated on a 5-point Likert scale, with response options ranging from “no problem” to “severe problems.” In addition, the scale comes with a visual analogue scale, which allows respondents to assess their overall health status by marking a point on a vertical, calibrated line that ranges from 0 (the worst imaginable health state) to 100 (the best imaginable health state).^[11]^

#### 2.5.3. Short-form health survey

The SF-12 has demonstrated reliability and validity in evaluating the HRQOL among individuals with CAD.^[22]^ The SF-12 has 8 domains: physical functioning, role physical, bodily pain, general health, vitality (VT), social functioning (SF), role emotional, and mental health (MH). The SF-12 questionnaire yields 2 summary scores: The physical component summary (PCS-12) score and the mental component summary (MCS-12) score. Scores range from 0 to 100, with higher scores indicating better physical and MH functioning.^[22]^

#### 2.5.4. Global rating of change scales

The GRC scale is used to assess a patient’s progress or deterioration over time. The GRC scale requires individuals to evaluate their present state of health, and report any changes in their health status over time since the last visit. The GRC scores have a scale from -5 to + 5, where a score of (-5) indicates a significant deterioration, a score of (+5) indicates a considerable improvement and a score of zero denotes no change. Patients with GRC scores ranging from −2 to 2 were considered unchanged for the test-retest reliability.^[23]^

### 2.6. Statistical analysis

All statistical analyses were performed using Statistical Package for Social Science version 26.0. (IBM Corp., Armonk). Descriptive statistics such as mean, standard deviation (SD), frequencies, and percentages were used to describe the data. The normal distribution of the data was evaluated using Shapiro–Wilk test.

### 2.7. Floor and ceiling effects

The SAQ-7 was evaluated for floor and ceiling effects. If over 15% of patients report the lowest score, it was concluded that the SAQ-7 has a floor effect. Similarly, if over 15% of patients report the highest score, the SAQ-7 was deemed to have a ceiling effect.^[20]^

### 2.8. Reliability

Internal consistency of the SAQ-7 was assessed using Cronbach alpha. In the current study, Cronbach alpha of 0.80 to 0.89 was considered very good, while a value of 0.90 or more was considered excellent internal consistency.^[24]^

Test-retest reliability of the SAQ-7 was examined using the ICC_2,1_ with 95% confidence intervals. The ICC_2,1_ was interpreted according to the following standards: weak (<0.50), moderate (0.50–0.75), good (0.75–0.90), and excellent (>0.90).^[24]^

### 2.9. Measurement error

The measurement error of the SAQ-7 was determined by calculating the standard error of measurement (SEM), which was computed as (SD × √1- ICC), where SD referred to the SD of the SAQ-7 scores and ICC refers to the test-retest reliability. The minimum detectable change (MDC) at a confidence interval of 95% was computed using the following formula (𝑀𝐷𝐶_95_=𝑆𝐸𝑀×1.96 × √2).^[24]^ The Bland and Altman analysis was used to assess the limits of agreements between the first and second test of the SAQ-7.^[25]^

### 2.10. Construct validity

Construct validity of the SAQ-7 was examined based on predefined hypotheses: The SAQ-7 would have at least moderate correlations (>0.50) with EQ-5D-5L and SF-12 scores.^[26]^ Both EQ-5D-5L and SF-12 were used to capture different perspectives on HRQOL. The EQ-5D-5L assesses overall health state, whereas SF-12 focuses on physical and MH functioning. The strength of the associations was interpreted according to the following guidelines: a correlation coefficient of 0.00 to 0.25 represents little or no relationship, 0.26 to 0.50 indicates a fair relationship, 0.51 to 0.75 indicates a moderate-to-good relationship and >0.75 indicates a good-to-excellent relationship.^[27]^

## 3. Results

### 3.1. Participant’s physical and clinical characteristics

A total of 258 participants were included in this study, comprising 76% males and 24% females, with a mean ± SD age of 54 ± 11 years. The participant’s demographic and clinical characteristics are detailed in Table 1. The majority of participants (70%) had hypertension, followed by hypercholesterolemia (65%) and diabetes (46%). Most participants had not participated in cardiac rehabilitation programs (79.5%), with 42.7% achieving education levels of a diploma or above.

**Table 1:** Baseline physical and clinical characteristics of participants.

Variable	N = 258, mean ± SD or n (%)
Male/female n (%)	196 (76%)/ 62 (24%)
Age (year)	54 ± 11
Weight (kg)	81.7 ± 13.5
Height (cm)	167.6 ± 8.7
BMI (kg/m^2^)	28.56 ± 4.7
Systolic blood pressure (mm Hg)	129 ± 13
Diastolic blood pressure (mm Hg)	80 ± 8
Educational level	
Elementary	30 (12%)
Intermediate	78 (30%)
High school	82 (32%)
Diploma or above	68 (26%)
Comorbidities	
Hypertension	107 (70%)
Hypercholesterolemia	85 (65%)
Type 2 Diabetes Mellitus	66 (46%)
EF (%)	50 (5)
SAQ-7 physical limitation	57.6 ± 30.7
SAQ-7 Angina frequency	73 ± 20.4
SAQ-7 quality of life	42.2 ± 29.3
SAQ-7 summary score	57.6 ± 22.5
EQ-5D-5L	10.2 ± 3.8
EQ-VAS	67.3 ± 17.5
PCS-12	38.6 ± 9.9
MCS-12	46.3 ± 12.1

BMI = body mass index, cm = centimeter, EF = ejection fraction, EQ_VAS = Euro-Quality visual analogue scale, kg = kilogram, m^2 =^ meter square, MCS = mental components summary, mm Hg = millimeters of mercury, n = number, N = total number of patients, PCS = physical components summary, SAQ = Seattle Angina Questionnaire, SD = standard deviation.

**Figure 1.**
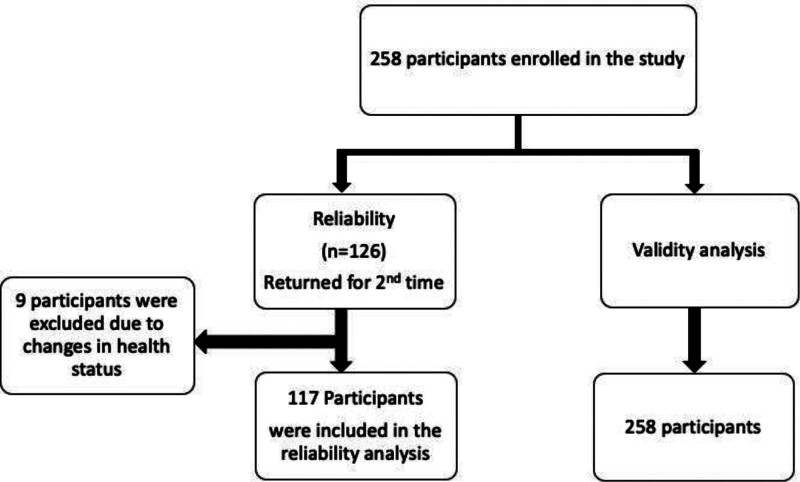
Flow diagram illustrating participant inclusion in reliability and validity analyses.

For the reliability analysis, 126 participants returned for the second visit. The GRC results indicated that 9 participants experienced significant changes in their health condition and were subsequently excluded from the analysis. As a result, the reliability analysis included a total of 117 participants (93 males) with a mean ± SD age of 52 ± 7 years (Fig. 1). The mean interval time between the first and second visits was 5 ± 1 days.

### 3.2. Floor and ceiling effects

The SAQ-7 revealed no significant floor or ceiling effects, as only 3% and 4% of participants achieved the lowest and highest scores, respectively. This indicates the tool’s sensitivity in capturing a wide range of HRQOL levels among patients post-MI.

### 3.3. Reliability

The SAQ-7 demonstrated a high level of internal consistency, as indicated by a Cronbach alpha value of 0.97 for the overall scale. Test-retest reliability was similarly high with an overall ICC_2,1_ of 0.94 (95% CI: 0.92–0.96, *P* <.001). The internal consistency and test-retest reliability of each domain of the SAQ-7 were also explored. The Physical Limitation domain showed excellent internal consistency (Cronbach alpha = 0.93) and reliability (ICC_2,1_ = 0.89, 95% CI: 0.85–0.92 *P* <.001). The Angina Frequency domain similarly showed excellent internal consistency (Cronbach alpha = 0.91) and reliability (ICC_2,1_ = 0.87, 95% CI: 0.82–0.91 *P* <.001). The Quality of Life domain also showed excellent internal consistency (Cronbach alpha = 0.91) and reliability (ICC_2,1_ = 0.90, 95% CI: 0.86–0.93 *P* <.001).

The SEM for the SAQ-7 was 1.76, with a MDC at 95% confidence (MDC_95%_) of 4.87 points. The Bland–Altman analysis showed a mean difference 0.75 between repeated measurement with narrow limits of agreement (-13.3 to 14.4).

### 3.4. Construct validity

Construct validity was assessed by correlating the SAQ-7 with other validated measures of HRQOL, including the EQ-5D-5L and SF-12. The physical limitation domain of the SAQ-7 exhibited moderate-to-excellent negative correlations with the EQ-5D-5L domains of Mobility (r = -0.76, *P* <.001), Self-Care (*r* = −0.69, *P* <.001), and Usual Activities (r = -0.68, *P* <.001), and a positive correlation with the SF-12 PCS (*R* = 0.64, *P* <.001). The angina frequency domain showed good correlations with the EQ-5D-5L pain/discomfort domain (*r* = −0.57, *P* <.001) and SF-12 PCS (*R* = 0.62, *P* <.001). The Quality of Life domain was significantly associated with the EQ-5D-5L anxiety/depression domain (*r* = −0.54, *P* <.001) and both the SF-12 PCS (*R* = 0.55, *P* <.001) and MCS (*R* = 0.55, *P* <.001). The overall SAQ-7 summary score demonstrated strong correlations with the EQ-5D-5L total score (*r* = −0.86, *P* <.001) and EQ-VAS (*R* = 0.66, *P* <.001), as well as the SF-12 PCS (*R* = 0.74, *P* <.001) and MCS (*R* = 0.57, *P* <.001).

## 4. Discussion

This study provides new evidence supporting the psychometric properties of the SAQ-7 in patients post-MI. The findings demonstrated high internal consistency, excellent test-retest reliability, and adequate construct validity, supporting the SAQ-7 as a reliable and valid scale for assessing the HRQOL of patients post-MI. The findings align closely with existing research while offering novel insights specific to the post-MI population.

Patient-centered care has increasingly emerged as a priority within healthcare systems, underscoring the importance of integrating PROMs as essential tools for capturing patients’ perspectives on their health status and monitoring their health trajectories over time. For this purpose, PROMs must be stable and reliable. In the current study, we observed no floor or ceiling effects of the SAQ-7. These findings are consistent with previous validations studies by in patients with CAD and ischemic heart disease.^[15,18]^ This finding reenforces the ability of the SAQ-7 to sensitively capture the full spectrum of HRQOL in patients post-MI, making it suitable for detecting fluctuations in health status, and monitoring changes over time.

The SAQ-7 showed excellent internal consistency and test-retest reliability across all 3 domains. High internal consistency across domains reflects strong homogeneity within the scale and confirms its diversity across different cultural backgrounds. Likewise, the excellent test-retest indicates stability over time. These results are consistent with those of the original SAQ-7 validation study by Chan et al, who reported strong internal consistency (Cronbach α ≥0.90), excellent test-retest reliability (ICC ≥0.78) over a 1 month interval in patients with CAD.^[16]^ In our study, reliability was even higher, with Cronbach α = 0.97 and ICC = 0.94, suggesting stronger internal consistency and temporal stability in a post-MI cohort. These higher reliability metrics may reflect the homogeneity of our study population and standardized data collection methods. Moreover, the interval period in our study was almost 1 week likely minimized changes in participants’ health status while preventing recall bias, thereby enhancing reliability.

A unique contribution of this study is the calculation of the SEM and MDC_95%_ for the SAQ-7, providing valuable insights into its sensitivity to detect meaningful changes in health status among patients post-MI. The observed SEM of 1.76 and MDC_95%_ of 4.87 fall within acceptable ranges, supporting the SAQ-7 capability to detect clinically meaningful changes. While prior metrics of the SAQ-7 are unavailable, previous international studies on the SAQ-19 have reported SEM values between 1.5 and 2.0 and MDC values around 4 to 5 points, which are consistent with our current findings.^[17–19]^ Additionally, this study uniquely further supported the reliability of the SAQ-7 by evaluating the LOA. The Bland and Altman analysis showed no systematic bias in scores with good agreement between repeated measurements. These findings further support the utility of the SAQ-7 in both clinical and research settings.

The association of the SAQ-7 with other validated scales for HRQOL, including the EQ-5D-5L and SF-12 confirmed its construct validity. In this study, we found that the SAQ-7 can capture different aspects in health status as other validated PROMs do. The angina frequency domain of the SAQ-7 showed moderate-to-good correlations with the EQ-5D-5L pain/discomfort domain and the SF-12 physical components. Similarly, the physical limitation domain of the SAQ-7 showed moderate to excellent negative correlations with the mobility, self-care, and usual activities domains of the EQ-5D-5L, and with PCS of the SF-12. These findings align with prior findings, such as Patel et al study, which reported similar associations between the physical limitation domain of SAQ-7 and SF-12 physical components in patients with acute MI.^[18]^ Moreover, the quality of life domain of the SAQ-7 also correlated with the EQ-5D-5L anxiety/depression domain, and the SF-12 physical and mental components. These findings further reinforce the ability of the SAQ-7 to capture comprehensive HRQOL dimensions.

Accurate evaluating of patients’ angina and overall health status is a key component of patient care during clinical visits. However, research shows that cardiologists often fail to accurately and consistently assess patients’ symptoms.^[28–30]^ Interesting, significant variability exists among cardiologists in identifying and documenting angina, with patients being twice as likely, on average, to have their angina under-recognized by 1 cardiologist compared to another.^[31]^ Underestimation of angina can delay appropriate treatment escalation, such as referral for revascularization, leading to persistent symptoms and reduced HRQOL. Conversely, overestimation may lead to unnecessary revascularization, exposing patients to potential risks that outweigh the benefits.^[32]^ Implementing a standardized HRQOL tool with consistent questions over time such as the SAQ-7 could help address these inconsistencies and enhance clinical decision making.

### 4.1. Limitations

The study had a sex imbalance, with most participants being male, which may impact the generalizability of the findings. Additionally, the assessment of responsiveness, which is a critical psychometric property, was not included. The SAQ-7 demonstrated excellent psychometric properties, confirming its reliability and validity for post-MI HRQOL assessment. However, these findings should be interpreted in the context of the study population and design. Future research should evaluate responsiveness and test applicability across diverse clinical settings.

## 5. Conclusion

The SAQ-7 demonstrated excellent psychometric properties, supporting its reliability, validity, and suitability for patients post-MI. The SAQ-7 may offer a practical, efficient, and sensitive method for evaluating the impact of MI on HRQOL, making it a valuable tool for both clinical practice and research. Additionally, its integration into routine clinical practice could support patient-centered care by facilitating the monitoring of health trajectories and guiding tailored interventions.

## Acknowledgements

The authors would like to thank Ongoing Research Funding program (ORF-2025-647), King Saud University, Riyadh, Saudi Arabia.

## Author contributions

**Conceptualization:** Ali Albarrati, Rakan Nazer.

**Data curation:** Taghreed Alotaibi.

**Formal analysis:** Ali Albarrati, Taghreed Alotaibi.

**Investigation:** Taghreed Alotaibi.

**Methodology:** Ali Albarrati, Taghreed Alotaibi, Rakan Nazer.

**Supervision:** Ali Albarrati, Rakan Nazer.

**Validation:** Taghreed Alotaibi.

**Writing – original draft:** Ali Albarrati, Taghreed Alotaibi.

**Writing – review & editing:** Rakan Nazer.

## References

[1] Brorsson B Bernstein SJ Brook RH Werkö L. Quality of life of patients with chronic stable angina before and four years after coronary revascularisation compared with a normal population. Heart. 2002;87:140–5. 11796552 10.1136/heart.87.2.140 PMC1766984

[2] Parikh KS Coles A Schulte PJ. Relation of angina pectoris to outcomes, quality of life, and response to exercise training in patients with chronic heart failure (from HF-ACTION). Am J Cardiol. 2016;118:1211–6. 27561194 10.1016/j.amjcard.2016.07.040 PMC5050169

[3] Arnold SV Morrow DA Lei Y. Economic impact of angina after an acute coronary syndrome. Circulation. 2009;2:344–53. 20031860 10.1161/CIRCOUTCOMES.108.829523

[4] Kawecka-Jaszcz K Klocek M Tobiasz-Adamczyk B Bulpitt CJ. Health-related quality of life in cardiovascular. Patients. 2013;1:1–133.

[5] Moryś JM Bellwon J Höfer S Rynkiewicz A Gruchała M. Quality of life in patients with coronary heart disease after myocardial infarction and with ischemic heart failure. Arch Med Sci. 2016;12:326–33. 27186176 10.5114/aoms.2014.47881 PMC4848348

[6] Sajobi TT Wang M Awosoga O; APPROACH Investigators. Trajectories of health-related quality of life in coronary artery disease. Circulation. 2018;11:e003661. 29545392 10.1161/CIRCOUTCOMES.117.003661

[7] Thompson DR Yu C-M. Quality of life in patients with coronary heart disease-I: assessment tools. Health Qual Life Outcomes. 2003;1:42. 14505492 10.1186/1477-7525-1-42 PMC201013

[8] Preedy VR Watson RR. Handbook of Disease Burdens and Quality of Life Measures. Springer; 2010:179–94.

[9] Brooks R. EuroQol: the current state of play. Health Pol. 1996;37:53–72. 10.1016/0168-8510(96)00822-6 10158943

[10] Nowels D McGloin J Westfall JM Holcomb S. Validation of the EQ-5D quality of life instrument in patients after myocardial infarction. Qual Life Res. 2005;14:95–105. 15789944 10.1007/s11136-004-0614-4

[11] De Smedt D Clays E Doyle F; EUROASPIRE Study Group. Validity and reliability of three commonly used quality of life measures in a large European population of coronary heart disease patients. Int J Cardiol. 2013;167:2294–9. 22748284 10.1016/j.ijcard.2012.06.025

[12] Patrick DL Deyo RA. Generic and disease-specific measures in assessing health status and quality of life. Med Care. 1989;27(3 Suppl):S217–32. 2646490 10.1097/00005650-198903001-00018

[13] Spertus JA Winder JA Dewhurst TA Deyo RA Fihn SD. Monitoring the quality of life in patients with coronary artery disease. Am J Cardiol. 1994;74:1240–4. 7977097 10.1016/0002-9149(94)90555-x

[14] Spertus JA Winder JA Dewhurst TA. Development and evaluation of the Seattle Angina questionnaire: a new functional status measure for coronary artery disease. J Am Coll Cardiol. 1995;25:333–41. 7829785 10.1016/0735-1097(94)00397-9

[15] Chan PS Jones PG Arnold SA Spertus JA. Development and validation of a short version of the seattle angina questionnaire. Circulation. 2014;7:640–7. 10.1161/CIRCOUTCOMES.114.000967 PMC4282595 25185249

[16] Maron DJ Hochman JS O’Brien SM; ISCHEMIA Trial Research Group. International study of comparative health effectiveness with medical and invasive approaches (ISCHEMIA) trial: rationale and design. Am Heart J. 2018;201:124–35. 29778671 10.1016/j.ahj.2018.04.011 PMC6005768

[17] Spertus J Mark D. ISCHEMIA trial update. Am Heart J. 2019;218:8. 31665620 10.1016/j.ahj.2019.09.001 PMC6927403

[18] Patel KK Arnold SV Chan PS. Validation of the Seattle angina questionnaire in women with ischemic heart disease. Am Heart J. 2018;201:117–23. 29772387 10.1016/j.ahj.2018.04.012 PMC6047765

[19] Arnold SV Kosiborod M Li Y. Comparison of the seattle angina questionnaire with daily angina diary in the TERISA clinical trial. Circulation. 2014;7:844–50. 10.1161/CIRCOUTCOMES.113.000752 25249560

[20] Terwee CB Mokkink LB Knol DL Ostelo RWJG Bouter LM de Vet HCW. Rating the methodological quality in systematic reviews of studies on measurement properties: a scoring system for the COSMIN checklist. Qual Life Res. 2012;21:651–7. 21732199 10.1007/s11136-011-9960-1 PMC3323819

[21] Marx RG Menezes A Horovitz L Jones EC Warren RF. A comparison of two time intervals for test-retest reliability of health status instruments. J Clin Epidemiol. 2003;56:730–5. 12954464 10.1016/s0895-4356(03)00084-2

[22] Failde I Medina P Ramirez C Arana R. Construct and criterion validity of the SF-12 health questionnaire in patients with acute myocardial infarction and unstable angina. J Eval Clin Pract. 2010;16:569–73. 20438603 10.1111/j.1365-2753.2009.01161.x

[23] Kamper SJ Maher CG Mackay G. Global rating of change scales: a review of strengths and weaknesses and considerations for design. J Manual Manipulative Ther. 2009;17:163–70. 10.1179/jmt.2009.17.3.163 PMC2762832 20046623

[24] Koo TK Li MY. A guideline of selecting and reporting intraclass correlation coefficients for reliability research. J Chiropractic Med. 2016;15:155–63. 10.1016/j.jcm.2016.02.012 PMC4913118 27330520

[25] Bland JM Altman DG. Statistical methods for assessing agreement between two methods of clinical measurement. Lancet. 1986;1:307–10. 2868172

[26] Terwee CB Bot SDM de Boer MR. Quality criteria were proposed for measurement properties of health status questionnaires. J Clin Epidemiol. 2007;60:34–42. 17161752 10.1016/j.jclinepi.2006.03.012

[27] Mokkink LB Terwee CB Patrick DL. The COSMIN study reached international consensus on taxonomy, terminology, and definitions of measurement properties for health-related patient-reported outcomes. J Clin Epidemiol. 2010;63:737–45. 20494804 10.1016/j.jclinepi.2010.02.006

[28] Shafiq A Arnold SV Gosch K. Patient and physician discordance in reporting symptoms of angina among stable coronary artery disease patients: insights from the Angina prevalence and provider evaluation of angina relief (APPEAR) study. Am Heart J. 2016;175:94–100. 27179728 10.1016/j.ahj.2016.02.015 PMC5266615

[29] Saxon JT Chan PS Tran AT. Comparison of patient-reported vs physician-estimated angina in patients undergoing elective and urgent percutaneous coronary intervention. JAMA Netw Open. 2020;3:e207406. 32558912 10.1001/jamanetworkopen.2020.7406 PMC7305522

[30] Qintar M Spertus JA Gosch KL. Effect of angina under-recognition on treatment in outpatients with stable ischaemic heart disease. Eur Heart J. 2016;2:208–14. 10.1093/ehjqcco/qcw016 PMC5322471 28239488

[31] Arnold SV Grodzinsky A Gosch KL. Predictors of physician under-recognition of angina in outpatients with stable coronary artery disease. Circulation. 2016;9:554–9. 27531922 10.1161/CIRCOUTCOMES.116.002781 PMC5031528

[32] Malik AO Spertus JA Patel MR Dehmer GJ Kennedy KF Chan PS. Potential association of the ISCHEMIA trial with the appropriate use criteria ratings for percutaneous coronary intervention in stable ischemic heart disease. JAMA Internal Med. 2020;180:1540–2. 32955575 10.1001/jamainternmed.2020.3181 PMC7506580

